# Supplementary material for: Comprehensive screening of low nitrogen tolerant maize based on multiple traits at the seedling stage
Source: PeerJ. 2022 Oct 18;10:e14218. doi: 10.7717/peerj.14218 (PMC9586120; doi:10.7717/peerj.14218)
Supplement: Supplemental Information 1 [file peerj-10-14218-s001.docx]

| **Table S1** Sequence of the primers used for real-time PCR | | |
| --- | --- | --- |
| **Gene** | **Accession No.** | **Primer** |
| *ZmAMT1;1a* | GRMZM2G175140 | F-5'-GTGGCGGGCTGCTGGTCAAGA-3' |
|  |  | R-5'-CGACCGTCAAAGCCGCTAGATTG -3' |
| *ZmAMT1;3* | GRMZM2G028736 | F-5'-GCAGTTCGTGGCGTACCTCATC-3' |
|  |  | R-5'-CACCATGTGGACAACGCTGGAC-3' |
| *ZmNRT2;1* | GRMZM2G010280 | F-5'-CGACGAGAAGAGCAAGGGACT-3' |
|  |  | R-5'-GGCATATTCGTACATACAAAGAGGT-3' |
| *ZmNRT2;2* | GRMZM2G010251 | F-5’-CGACGAGAAGAGCAAGGGACT-3’ |
|  |  | R-5’-AGGTGAACATGGATGATGGAT-3’ |
| *Zm**GAPDH* | GRMZM2G046804 | F-5'-CTGGTTTCTACCGACTTCCTTG-3' |
|  |  | R-5'-CGGCATACACAAGCAGCAAC-3' |
